# Influence of Formal Education on Cognitive Reserve in Patients with Multiple Sclerosis

**DOI:** 10.3389/fneur.2016.00046

**Published:** 2016-03-29

**Authors:** Ralf Luerding, Sophie Gebel, Eva-Maria Gebel, Susanne Schwab-Malek, Robert Weissert

**Affiliations:** ^1^Department of Neurology, University of Regensburg, Regensburg, Germany

**Keywords:** multiple sclerosis, neuropsychology, cognition, cognitive reserve, educational status

## Abstract

The concept of cognitive reserve (CR) and its influence on cognitive impairment has attracted increasing interest. One hundred twenty-eight patients with multiple sclerosis (MS) from Southern Germany were evaluated during the years 2000 to 2012. Twenty-seven neuropsychological (NP) tests were applied regarding basic cognitive functions, attention, executive functions, visual perception and construction, memory and learning, problem solving, and language. By this retrospective approach, a comprehensive NP profile of the investigated individuals was established. An effect of timespan of formal education on CR was observed. Enrichment by reading, physical activities, and challenging vocational practices had more profound effects in patients who had undergone a shorter educational period compared to a longer educational period. In summary, our study demonstrates that the advantage of longer formal education periods, compared to shorter formal education periods, can be counterbalanced by high frequencies of reading, physical activities, and challenging vocational practices in patients with MS.

## Introduction

Multiple sclerosis (MS) is a demyelinating and neurodegenerative disease of the central nervous system (CNS) with both white matter and cortical lesions, as well as brain atrophy (1, 2). About 85% of MS patients initially present with a relapsing–remitting type of MS (RRMS) with acute disease exacerbations and remissions. Within 10 years, about 50% of patients progress to a secondary chronic progressive type of MS (SPMS) with progressive deterioration of neurological functions (3). Up to 15% of MS patients have primary progressive MS (PPMS) with steadily increasing disability. With its particular clinical and pathological spectrum, it is not surprising that it is estimated that 43–65% of patients with MS exhibit symptoms of cognitive impairment (CI) and decline at some point during their disease (4, 5). CI describes the grade and the quality of slowing or loss-of-functions, such as memory, attention, language, visuoperception, and visuoconstruction. Lately, the concept of cognitive reserve (CR), initially developed and introduced in the context of Alzheimer’s disease (AD) (6–10), has been extended and investigated in patients with MS (11–14). The concept of CR includes all phases of activities before the disease, in the beginning of the disease, and in the course of the disease (12). A study on CR has to select one specific phase of activities relevant for CR for focusing on the effects of this specific phase. Our study was not designed to examine postdisease effects or treatment but the phase before the onset and in the beginning of the disease. Usually, *passive* and *active* components of CR are differentiated. *Passive* reserve focuses on the brain’s structural accouterment, such as neuronal count, number of synapses, overall size, and cortical thickness, in dealing with neuronal damage and is sometimes referred to as “brain reserve capacity” (BRC) (5, 15, 16). It provides information on how much damage can be sustained before a threshold for clinical manifestation is reached. *Active reserve* refers to different, individualized processes and strategies that the brain actively implements to cope with neuronal damage (16, 17). Studies designed to investigate this context have used various proxy measures, such as in the case of *active* CR, most commonly educational level, occupational characteristics, as well as both physical and cognitively stimulating leisure activities (18). Partly, the concepts are not fully explored, especially in the case of *passive* CR due to technical limitations, namely, that the anatomical basis and the relationship to its cognitive substrate are not fully understood. Moreover, even with the active reserve, some research questions still need to be solved. In particular, the applicability of study findings pertaining to different cultural systems, in which the school system, professional life, and language can vary extensively, thus possibly influencing the proxy measures for CR in clinical studies. This is especially true for educational background, reading, and occupation. A longer time of formal education can lead to a higher CR, because there is more time to collect skills, which can be used for compensation of reduction of certain cognitive functions (19–22). There is the question if patients with a shorter period of formal education can profit from factors of CR, because this group had not the opportunity to collect the same amount of skills for compensation compared to a group with a longer period of formal education. In this retrospective study, the different effects from factors of CR were analyzed in two groups of patients with the shortest time of formal education compared to the group with the longest time of formal education. It would be counterintuitive if the patients with the shortest period of formal education would reach the same level of effects compared to the patients with the longest period of formal education.

## Materials and Methods

### Ethics

The patient data were collected on a routine basis after specific request by the treating MS physician. The NP examination was performed by a neuropsychologist. The data were collected on paper scoring sheets with a standardized protocol and put into the hardcopy patient file. In addition, data regarding disease course and data for the estimation of premorbid performance level were collected. Subsequently, the NP testing scores and this data were put in a collection file into a database by an independent investigator. Then, data were anonymized. The legislation is based on the German Code of Medical Ethics §15. In accordance with this, the Institutional Review Board (IRB) of the University of Regensburg approved this retrospective epidemiological study (Ethical permission No. 14-101-0050). Patients were informed about the subject and goal of the examination and consented on the clinical procedures.

### Patient Selection

Southern German Patients with MS, who were treated at the Department of Neurology of the University of Regensburg during the years 2000 to 2012, were investigated and evaluated in terms of general personal information, marital status, educational background, current work status, nicotine, liquor and drug abuse, family history concerning MS, clinical onset, diagnosis, type of disease (RRMS, SPMS, and PPMS), administered medication, comorbidities, and clinical parameters relating to the medical condition, such as magnetic resonance imaging (MRI), neurophysiological and neuropsychological (NP) test results, and extended disability status scale (EDSS) scores based on the Kurtzke model (23). Patients who reported to have suffered from a subjective disturbance of cognitive performance were assessed by the treating neurologist of the Department of Neurology of the University of Regensburg for NP testing.

### The Neuropsychological Test Battery

A NP examination measures in individuals the performance of memory, attention, language, visuoconstruction, and visuoperception with standardized tests that are validated though lesion studies for a measurement of neuronal functions in comparison to healthy controls of the same age group. The NP test battery, with a duration of about 2–3 h in a unique session performed by two expert neuropsychologists, consisted of measures of basic cognitive functions, attention, executive functions, visual perception and construction, memory and learning, as well as problem solving and language (detailed Table 1; Table S1 in Supplementary Material). Test scores were evaluated based on standard procedures and transformed to *z*-scores for statistical analysis in order to be able to compare each patient’s scores with a healthy sample group of the same age. The high number of tests was selected in order to attain a comprehensive picture of the patients’ cognitive performance in regard to multiple NP subsystems.

**Table 1:** **Overview of subsystems and used tests**.

Subsystems	Tests
Basic cognitive functions (BRC)	● WAIS-R
	○ Information
	○ Comprehension
	○ Similarities
	○ Digit symbol test
	○ Picture completion
	○ Block design
Attention	● Digit symbol test (WAIS-R)
	● Trail making test B (TMT-B)
	● TAP (test battery for attention)
	○ TAP RWOW: reaction time without warning
	○ TAP ROW: reaction time with warning
	○ TAP PA: phasic alertness
	● Ruff 2 and 7
	○ Numbers
	○ Letters
	○ Performance
Executive functions	● Similarities (WAIS-R)
	● Block design (WAIS-R)
	● Picture completion (WAIS-R)
	● Digit symbol test (WAIS-R)
Visual perception and construction	● Trail making test A (TMT A)
	● Picture completion (HAWIE-R)
	● Block design (WAIS-R)
	● Rey complex figure test (RCFT) copy
Memory and learning	● Digit span (DS) forwards and backwards
	● Corsi blocks
	● California verbal learning test (CVLT)
	○ Performance
	○ Recognition
	● Wechsler memory scale (WMS)
	○ Immediate recall
	○ Delayed recall
	○ Recognition
	● Rey complex figure retention test (RCFT)
	○ Performance
	● Rey visual design verbal learning test (RVDLT)
Problem solving and language	● Similarities (WAIS-R)
	● Controlled oral word association test (COWA)
	● Semantic verbal fluency (SVF)

*All tests were used in German language and are validated*.

The test battery was composed for a sensitivity unrelated to a specific disease, but for a sensitivity for impairments for all neurological diseases. This allows comparison of impairments to concurrent etiologies of MS. The test battery has been used in several studies (24–27).

The NP assessment was standardized to a fixed protocol over all patients, so the assessment did not differ in this retrospective study from an assessment of a prospective study.

### Educational Background

The grouping of patients, regarding educational background, was based on the German education system that is unique and cannot be transferred and directly compared to countries with other schooling systems, such as the United Kingdom or the United States of America. Patients were divided into three different educational groups based on the years of school that they had attended and the highest diploma or degree they had attained during the educational process. Consequently, patients in group 1 had left school after 9 years or less, without or with the degree of Hauptschule (school from year 5 to 9 in Germany), whereas patients in group 2 had graduated after 10 years of school with a Realschule certificate. Finally, group 3 consisted of patients that attended school or institutions for higher-leveled education for at least 13 years and had graduated with the Abitur (general qualification for University entrance in Germany) or in addition received a University level diploma afterwards. In this study, the educational background is informative on the premorbid performance level, since the beginning of disease is in nearly all cases later than the termination of School and University.

### Occupation

Concerning occupational status, patients were split into three different groups based on the level of independent working and thinking, abstract-logical requirements that their reported job demanded of them, and the degree of vocational school. Consequently, group 1 are patients with no vocational education or patients who attended vocational school, whereas group 2 comprises patients who have a higher vocational degree compared to group 1, and whose jobs demand more abstract and independent thinking. Group 3, in turn, comprises patients who graduated from Polytechnics or Universities and whose jobs demand the highest degree of independence and abstract thinking. The information on occupation is also an estimation of a premorbid performance level for the same reason as the educational background.

### Physical Activity

Physical activity of the patient cohort was evaluated based on the amount of time spent on exercise and its regularity, as well as the specific type of exercise exerted. In order to do so, patients were divided into four groups. Group 0 consisted of patients who do not do physical exercise present nor in the past. Group 1 included patients who stopped formally to perform sports, but in the past had practiced challenging sports with a regular pattern, and those who reported that they were only sporadically doing those types of physical exercise that demand little physical effort, such as walking or physical rehabilitation. Patients in group 2 did less strenuous kinds of exercise, such as walking or physical rehabilitation, but on a regular basis. Patients in group 3 engaged in regular, challenging physical exercise, such as running or swimming, at least once a week. The information on physical activity is a mixture of premorbid and actual performance level. Physical exercise, without characteristics of rehabilitation therapy, was started in most cases before the beginning of disease. Only exercise with rehabilitation characteristics would be counted as information on actual performance.

### Reading Activity

For statistical analysis in terms of reading activity, we split the examined patients into three different groups. Patients in group 0 reported to be doing no reading or to be reading only rarely. Patients’ reading in group 1 consisted of non-challenging material, such as the sports column in the newspaper or magazines and was performed on a regular or non-regular basis; furthermore, it included those patients whose reading involved challenging material, such as professional journals, long novels, or the whole newspaper, but was not done regularly. Patient group 2 included those patients that engaged in regular, or even daily, reading of challenging material at least once a week. Since reading is a habit strongly dependent on the frequency in daily living, it is quite implausible that frequent reading is started after the beginning of disease. Therefore, a high frequency in reading is a clear sign for a premorbid performance level.

### Statistics

Since the information on educational and professional background, physical, and reading activity was scaled non-parametrically, we used statistical tests compatible with data possessing those properties. We hypothesized that, in accordance with results found for Alzheimer’s dementia and previous reports on studies with MS patients, education, occupation, and physical and reading activity were connected with patients’ cognitive performance on NP testing. In order to examine the relation between cognition and the leisure and job activities, we used Mann–Whitney *U*-tests that compared two of the groups. For correction for multiple testing, we applied the Bonferroni method. Consequently, the level of significance was determined to be *p* = 0.002. Twenty-five comparisons were computed. Effects not surviving the Bonferroni correction were reported nonetheless because of the risk of underestimation of effects, since the Bonferroni correction is rigorous.

All NP test scores were transformed in *z*-scores (raw scores − mean scores of age group/SD of the age group) for correction of age effects. Univariate analyses of variances were computed for the group of patients with the shortest period of education with the factors reading, physical activity, and occupation for effects on cognitive performance. This procedure was repeated for the group of patients with the longest period of education, in order to discriminate effects of CR from an advantage of acquiring skills for compensation through education. Patients with a moderate time of education were not included in this ANOVA. For all statistical analysis, PASW Statistics 18 (formerly SPSS Statistics) was used.

## Results

### Patients

One hundred twenty-eight MS patients were included in our study, of which 81 (63.3%) were females and 47 (36.7%) were males. Mean age of the cohort was 48 years (minimum 20, maximum 86) with mean disease duration being 6.75 years (minimum 0 years, maximum 31 years) and media EDSS 4.0 (range 0–9.0). Sixty-six (51.6%) patients presented with RRMS, 34 (26.6%) with SPMS, 21 (16.4%) with PPMS, and 7 (5.4%) with other types, which included 5 patients with a clinically isolated syndrome (CIS) that later developed MS, 1 patient with acute disseminated encephalomyelitis (ADEM), and 1 patient with opticospinal MS that later proved to be neuromyelitis optica (NMO) (Table 2). In terms of educational background, 24 patients (18.8%) had acquired Abitur or higher University degrees (group 3), 40 (31.3%) attended Realschule (group 2), and 64 (50%) had attended Hauptschule (group 1) (Table 2).

**Table 2:** **Characteristics of MS patients**.

Patient characteristics		
	**Number (*n*)**	**%**

Female	81	63.3
Male	47	36.7
RRMS	66	51.6
SPMS	34	26.6
PPMS	21	16.4
Others	7	5.5
13 years of formal education (Abitur)	24	18.8
10 years of formal education (Realschule)	40	31.3
5–9 years of formal education (Hauptschule)	64	50

	**Years (mean)**	**Range**

Age	48	20–86
Disease duration	6.75	0–31
EDSS (median)	4	0–9.0

### Cognition and Education

Educational background of patients with MS, which was evaluated based on the three different educational groups, was connected to CI in all NP subsystems (*p* = 0.002) and visual perception and construction (*p* = 0.05) (Table 3). In general, patients with a higher educational level scored better test results than those with a lower level. The detected group differences manifested, especially upon comparing patients with the highest educational background, i.e., group 3, with those patients with the two lower educational levels, i.e., groups 2 and 1, whereas the comparison between groups 1 and 2 only uncovered deviations regarding verbal memory skills. After Bonferroni correction, significant effects remained in the comparisons for immediate (*p* = 0.000; *M*_1_ = −0.883; *M*_3_ = 0.113) and delayed (*p* = 0.001; *M*_1_ = −0.979; *M*_3_ = −0.296) recall in the comparison of groups 1 and 3 and immediate recall for analysis of groups 2 and 3 (*p* = 0.002; *M*_2_ = −0.597; *M*_3_ = 0.113), as well as the results for similarities in both group contrasts between 1 and 2 (*p* = 0.001; *M*_1_ = −1.083; *M*_2_ = −0.351) and 1 and 3 (*p* = 0.001; *M*_1_ = −1.083; *M*_3_ = −0.035). The same applied to the results for comprehension (*p* = 0.000; *M*_1_ = −0.947; *M*_2_ = −0.044) and information (*p* = 0.003; *M*_1_ = −0.783; *M*_2_ = −0.021) between groups 1 and 2 and groups 1 and 3 (*p* = 0.001; *M*_1_ = −0.783; *M*_3_ = 0.191; *p* = 0.001; *M*_1_ = −0.947; *M*_3_ = −0.021), respectively. Consequently, these results imply an especially strong relation of educational background to learning and memory, executive functions, basic cognitive functions, problem solving, and language.

**Table 3:** **Differences in cognition according to formal educational level**.

Test name	*p*-Value	Mean values
**Group 1 versus group 2**		
California verbal learning test	0.007	*M*_1_ = −1.307
		*M*_2_ = −0.320
RVDLT	0.037	*M*_1_ = −1.738
		*M*_2_ = −1.275
Trail making test B	0.007	*M*_1_ = −1.616
		*M*_2_ = −0.819
Similarities	0.001	*M*_1_ = −1.083
		*M*_2_ = −0.351
Comprehension	0.000	*M*_1_ = −0.947
		*M*_2_ = −0.044
Information	0.003	*M*_1_ = −0.783
		*M*_2_ = −0.021
**Group 1 versus group 3**		
Wechsler memory scale: immediate recall	0.000	*M*_1_ = −0.883
		*M*_3_ = 0.113
Wechsler memory scale: delayed recall	0.001	*M*_1_ = −0.979
		*M*_3_ = −0.296
Trail making test B	0.034	*M*_1_ = −1.616
		*M*_3_ = −0.914
Digit symbol test	0.005	*M*_1_ = −0.931
		*M*_3_ = −0.257
Similarities	0.001	*M*_1_ = −1.083
		*M*_3_ = −0.035
Semantic fluency	0.041	*M*_1_ = −0.734
		*M*_3_ = −0.074
Information	0.001	*M*_1_ = −0.947
		*M*_3_ = −0.021
Comprehension	0.001	*M*_1_ = −0.783
		*M*_3_ = 0.191
**Group 2 versus group 3**		
Wechsler memory scale: immediate recall	0.002	*M*_2_ = −0.597
		*M*_3_ = 0.113
Delayed recall	0.001	*M*_1_ = −0.979
		*M*_3_ = −0.296

*Group 1 comprises patients who completed “Hauptschule,” group 2 comprises patients who completed “Realschule,” and group 3 comprises patients who completed higher German educational levels. Only test results with statistical significance are shown. Significance *p* < 0.05*.

### Cognition and Occupation

In terms of profession, it was detected that patients who had undertaken jobs that demand a high level of independency and abstract-logical thinking could achieve better results in all six cognitive subsystems. The effects were more pronounced between the group with the lowest level, concerning those demands, and those patients with a medium level, i.e., groups 1 and 2, while they decreased strongly when comparing the medium level with the high-level group, i.e., groups 2 and 3. The corresponding *p* values mostly displayed statistical significance based on an α-level of *p* = 0.05 (Table 4). This context requires special attention to be paid to the results of the information test (*p* = 0.001; *M*_1_ = −0.719; *M*_3_ = 0.364), in the comparison of groups 1 and 3, that indicate a durable and robust connection between basic cognitive function and cognition with the mean values giving better test scores for patients in group 3.

**Table 4:** **Differences in cognition according to occupation characteristics**.

Test name	*p*-Value	Mean values
**Group 1 versus group 2**		
Wechsler memory scale: immediate recall	0.025	*M*_1_ = −0.750
		*M*_2_ = −0.158
RVDLT	0.009	*M*_1_ = −1.717
		*M*_2_ = −0.862
Block design	0.029	*M*_1_ = −0.681
		*M*_2_ = −0.028
Similarities	0.019	*M*_1_ = −0.760
		*M*_2_ = −0.033
Information	0.013	*M*_1_ = −0.056
		*M*_2_ = 0.050
**Group 1 versus group 3**		
Digit span forwards	0.046	*M*_1_ = −0.721
		*M*_3_ = −0.250
Picture completion	0.018	*M*_1_ = −1.152
		*M*_3_ = −0.357
Information	0.001	*M*_1_ = −0.719
		*M*_3_ = 0.364
Comprehension	0.016	*M*_1_ = −0.506
		*M*_3_ = 0.307
**Group 2 versus group 3**		
Trail making test A	0.048	*M*_2_ = −0.547
		*M*_3_ = −1.457

*Group 1 comprises patients with no vocational education or patients who attended vocational school. Group 2 comprises patients who have a higher vocational degree compared to group 1 and whose jobs demand more abstract and independent thinking. Group 3 comprises patients who graduated from Polytechnics or Universities and whose jobs demand the highest degree of independence and abstract thinking. Only test results with statistical significance are shown. Significance *p* < 0.05*.

### Cognition and Physical Activity

A significant association was discovered in all six subsystems regarding cognition and physical activity (*p* = 0.05): patients who spent more time on physical activity scored better test results. The strongest effect was demonstrated between groups 0 and 3. The comparison of groups 2 and 3 indicated that patients whose exercise included a strenuous and challenging work-out routine, such as running or swimming, could obtain an additional effect. After Bonferroni correction, the comparison of patients in groups 2 and 3 (*p* = 0.001; *M*_2_ = −1.183; *M*_3_ = −0.210) remained significant (significance level of *p* = 0.002) (Table 5).

**Table 5:** **Differences in cognition according to physical activity**.

Test name	*p*-Value	Mean values
**Group 0 versus group 1**		
Information	0.041	*M*_0_ = −0.975
		*M*_1_ = −0.058
**Group 0 versus group 2**		
California verbal learning test	0.048	*M*_0_ = −1.082
		*M*_2_ = −0.522
**Group 0 versus group 3**		
Wechsler memory scale: immediate recall	0.009	*M*_0_ = −1.024
		*M*_3_ = −0.396
Wechsler memory scale: delayed recall	0.009	*M*_0_ = −1.081
		*M*_3_ = −0.578
Digit span forwards	0.016	*M*_0_ = −1.305
		*M*_3_ = −0.718
Digit span backwards	0.017	*M*_0_ = −1.380
		*M*_3_ = −0.838
Rey complex figure copy	0.006	*M*_0_ = −2.010
		*M*_3_ = −1.211
Semantic fluency	0.027	*M*_0_ = −0.875
		*M*_3_ = −0.021
**Group 1 versus group 2**		
Comprehension	0.030	*M*_1_ = −0.033
		*M*_2_ = −0.996
**Group 1 versus group 3**		
Digit symbol test	0.025	*M*_1_ = −1.182
		*M*_3_ = −0.348
**Group 2 versus group 3**		
Wechsler memory scale: delayed recall	0.034	*M*_2_ = −1.008
		*M*_3_ = −0.578
Rey complex figure retention	0.039	*M*_2_ = −1.125
		*M*_3_ = −0.712
Digit symbol	0.026	*M*_2_ = −1.074
		*M*_3_ = −0.348
Trail making test A	0.013	*M*_2_ = −1.755
		*M*_3_ = −0.866
Similarities	0.008	*M*_2_ = −1.159
		*M*_3_ = −0.323
COWA	0.024	*M*_2_ = −1.039
		*M*_3_ = −0.327
Semantic fluency	0.001	*M*_2_ = −1.183
		*M*_3_ = −0.210
Comprehension	0.008	*M*_2_ = −0.996
		*M*_3_ = −0.088

*Group 0 comprises patients who do and did not engage in physical activity. Group 1 comprises patients who used to do physical activity on a regular basis and those who currently engage only sporadically in physical activity. Group 2 comprises patients who do moderately strenuous types of physical activities on a regular basis. Group 3 comprises patients who engage in demanding physical activities on a regular basis. Only test results with statistical significance are shown. Significance *p* < 0.05*.

### Cognition and Reading Activity

Reading activity was correlated with basic cognitive functions, executive functions, visual perception and construction, and attention (*p* = 0.05) (Table 6). Upon comparing groups 1 and 2, it was discovered that those patients who read challenging material on a regular basis had an extra benefit in terms of learning and memory and problem solving and language.

**Table 6:** **Differences in cognition according to reading activity**.

Test name	*p*-Value	Mean values
**Group 0 versus group 1**		
Digit symbol test	0.049	*M*_0_ = −1.121
		*M*_1_ = −0.589
**Group 0 versus group 2**		
Digit symbol test	0.008	*M*_0_ = −1.121
		*M*_2_ = −0.383
Rey complex figure copy	0.030	*M*_0_ = −1.524
		*M*_2_ = −0.958
Block design	0.032	*M*_0_ = −0.676
		*M*_2_ = −0.047
Picture completion	0.050	*M*_0_ = −1.050
		*M*_2_ = −0.522
Information	0.035	*M*_0_ = −0.800
		*M*_2_ = −0.239
Comprehension	0.000	*M*_0_ = −0.843
		*M*_2_ = 0.289
**Group 1 versus group 2**		
California verbal learning test	0.026	*M*_1_ = −1.242
		*M*_2_ = −0.367
Recognition RVDLT	0.037	*M*_1_ = −1.636
		*M*_2_ = −1.542
Corsi blocks	0.017	*M*_1_ = −0.884
		*M*_2_ = −0.378
Block design	0.026	*M*_1_ = −0.667
		*M*_2_ = −0.047
Picture completion	0.007	*M*_1_ = −1.294
		*M*_2_ = −0.522
Similarities	0.009	*M*_1_ = −0.181
		*M*_2_ = −0.124
Comprehension	0.005	*M*_1_ = −0.472
		*M*_2_ = 0.289

*Group 0 comprises patients who are not doing any reading or who only do sporadic reading. Group 1 comprises patients who are only reading non-challenging material on a regular basis and those who read highly challenging material on a non-regular basis. Group 2 comprises patients who read challenging material on a regular basis at least once a week. Only test results with statistical significance are shown. Significance *p* < 0.05*.

### Influence of Timespan of Education on Cognitive Reserve

In the group 1 with the shortest time of education (*n* = 64), there were significant effects from occupational activities in non-verbal long-term memory (Table 7). An ambitious occupation was associated with a better performance in learning non-verbal designs (RVDLT 1–5) compared to patients in this group with more simple occupation. Effects were also found in this group, when patients reported a higher degree in reading activities for measures of visuoconstruction (block design WAIS-R, RCFT), verbal performance (similarities, comprehension, knowledge, WAIS-R), and non-verbal long-term memory (RCFT delay, RVDLT recognition) compared to patients of this group with low reading activity. Patients with the shortest time of education, which reported high frequency of physical activity, performed better in non-verbal long-term memory (RCFT delay) than patients with no or low physical activity.

**Table 7:** **Influence of short duration of formal education on CR**.

Factors	Neuropsychological tests	Domain	Mean values	*p*-Value
Physical activity	RCFT delay	Non-verbal long-term memory	*M*_0_ = −0.99	0.004
			*M*_1_ = −2.23	
			*M*_2_ = −1.24	
			*M*_3_ = −0.62	
Occupation	RVDLT trials 1–5	Non-verbal long-term memory	*M*_1_ = −1.90	0.010
			*M*2_*_	
			*M*_3_ = 0.15	
Reading	Similarities (WAIS-R)	Verbal reasoning	*M*_0_ = −1.57	0.008
			*M*_1_ = −1.17	
			*M*_2_ = −0.37	
	Verbal comprehension (WAIS-R)	Verbal reasoning	*M*_0_ = −1.42	0.002
			*M*_1_ = −0.95	
			*M*_2_ = −0.02	
	Knowledge (WAIS-R)	Verbal performance	*M*_0_ = −1.56	0.007
			*M*_1_ = −0.93	
			*M*_2_ = −0.49	
	Block design (WAIS-R)	Visuoconstruction	*M*_0_ = −1.13	0.040
			*M*_1_ = −0.80	
			*M*_2_ = −0.08	
	RCFT copy	Visuoconstruction	*M*_0_ = −1.90	0.008
			*M*_1_ = −1.29	
			*M*_2_ = −0.73	
	RVDLT recognition	Non-verbal long-term memory	*M*_0_ = −0.34	0.024
			*M*_1_ = −1.23	
			*M*_2_ = 0	

Significant differences of the univariate ANOVA in the patient group 1 (shortest time of education *n* = 64) in the factors physical activity, occupation, and reading activity on cognitive performance. *For the factor occupation, no patient was assigned to the group 2 (*n* = 0).

Patients with the longest time of education (group 3) performed better in non-verbal long-term memory, when they had a challenging occupation in comparison to patients of this group with more simple occupations (Table 8). Patients with the longest time of education performed better in attention (TMT-B), verbal long-term memory (CVLT trials 1–5), visuoconstruction (block design from WAIS-R), and verbal performance (comprehension from WAIS-R), when they reported a higher frequency of reading activity compared to patients of this group with a low frequency of reading activity. For the group with the longest time of education, there were no effects from physical activity on cognitive performance.

**Table 8:** **Influence of long duration of formal education on CR**.

Factors	Neuropsychological tests	Domain	Mean value	*p*-Value
Physical activity				No significant effects
Occupation	RCFT delay	Non-verbal long-term memory	*M*_1_ = −1.2	0.037
			*M*_2_ = −0.33	
			*M*_3_ = −1.0	
Reading	TMT-B	Attention	*M*_0_ = −2.14	0.045
			*M*_1_ = −0.61	
			*M*_2_ = −0.55	
	Block design (WAIS-R)	Visuoconstruction	*M*_0_ = −0.67	0.027
			*M*_1_ = −0.62	
			*M*_2_ = −0.43	
	Comprehension (WAIS-R)	Verbal performance	*M*_0_ = −0.42	0.047
			*M*_1_ = −1.83	
			*M*_2_ = −0.72	
	CVLT trials 1–5	Verbal long-term memory	*M*_0_ = −1.0	0.028
			*M*_1_ = −1.2	
			*M*_2_ = −0.92	

*Significant differences of the univariate ANOVA in the patient group 3 (longest time of education, *n* = 24) in the factors physical activity, occupation, and reading activity on cognitive performance*.

## Discussion

In this study, we demonstrate an influence of the duration of the period of formal education on CR in patients with MS. The group with the most limited period of formal education demonstrated the greatest effects of better cognitive performance through reading, physical activity, and challenging occupations. The group with the longest period of formal education did not show an effect from physical activity on cognition. The number of significant effects in the group with the longest time of formal education reached five measurements, whereas the group with the shortest time of formal education presented effects from reading, sports, and occupational strain, in seven measurements.

Because the effects of reading, physical activity, and occupation were stronger in the group with the shortest time of formal education, it can be assumed that in this group significant differences in cognitive performance are reached earlier than in the group with the longest time of formal education. The reason can be that the capabilities of neurocognitive performance are not used in the premorbid state to the possible extent. This would also explain that in the group with the longest time of formal education, the effects are smaller because the capability of neurocognitive performance is used and trained more adequately.

In the analysis on cognition and education over all groups, there were effects on cognitive performance through time of education, showing that a longer time of education was associated with better neurocognitive performance. This finding would support the assumption that a longer time of education would lead to a higher level of CR, because more skills for compensation can be collected. The difference between both extremities of time of education, in effects from factors of CR, support the assumption that the advantage of a longer education can be counterbalanced by high frequencies in reading, physical activities, and challenging vocational practices.

We show that CI, in all six subsystems of cognitive abilities of patients suffering from MS, is positively influenced by CR. Consequently, we were able to replicate the results of a simultaneously conducted study that proxies of CR apply to MS as well (11, 28).

A limitation in interpreting the data on cognition are confounds of education, occupation, and reading on the cognitive performance. The influence of these data on cognition is reciprocal, so a higher score in education, occupation, and reading can result in a positive training effect in measures of cognition. Without an intact neurocognitive base before starting the career of education and occupation, the influence on training effects through education, occupation, and reading is not conceivable. To reach a higher grade in education or/and occupation, there has to be an average level of cognitive performance at minimum. For stabilizing a higher level in cognitive performance, a higher complexity in occupation is necessary for longer periods. A higher degree on each of the both sides is not likely without one of the other.

In addition, the study was able to establish that different parameters of CR mediated the impact of CI in patients with MS. These observations are concordant with the observation that leisure activities independently improve cognitive status as well as brain atrophy (22) and that the CR theory is therefore extendable to MS (28). Moreover, the results of our study comport with previous and more extensive results published on this topic in context with AD, which have detected a positive influence of educational level (6, 18), occupation (6), and physical activity (10, 29) as well as reading activity (8, 9) on CI. Consequently, just like in AD, CI in patients with MS seems to be positively influenced and mediated by CR.

Limitations of our study are that the assessment was not done according to a prospective protocol. Presently, the retrospective approach is the only methodological feasible study type to describe effects of activities before the onset and in the beginning of the disease in higher numbers of patients. In addition, the overall assessment would not have differed and was equally performed by two expert neuropsychologists in all patients. Another limitation could also be the fact that the presented results might not be applicable to different countries since the German schooling system is unique and selects and groups children and adults more cohesively and comprehensively in regard to preexisting cognitive abilities. Yet, this in turn is also a strength of this study because it contributes to a bigger and fuller picture of cognitive performance of the MS patients overall, and especially in Germany, thus helping with improving and specifying exciting problems with the CR theory, especially in the case of the active reserve. Due to the type of the study, we did not analyze MRI data in this study. It has been shown that in CI in MS patients the topography of lesions is more important than the number of lesions (30). A small number of lesions in critical localizations influence cognitive performance more than a higher number of lesions in non-critical localizations.

The study is important in terms of new therapeutic strategies for MS patients exhibiting cognitive symptoms, since it uncovers different aspects how cognition can be maintained and improved in a setting that differs very much from those found in other countries. Together with future research on the topic, the results will hopefully contribute to establish a more specified and comprehensive picture of how CR proxies are influenced by different cultural systems and to design and develop behavioral programs and rehabilitation recommendations for cognitively impaired patients with MS.

## Conclusion

In conclusion, our study supports the importance of CR on the degree of CI in patients with MS and also the applicability of the theory of CR to a German cohort with different social, school, and language backgrounds. One of the most interesting findings of this study was the difference between the groups with the shortest and the longest time of formal education: the group with the shortest time of formal education could profit from factors of CR more than the group with the longest time of formal education. So, the results can be interpreted as a hint that an advantage through a longer formal education might be counterbalanced by activities independent from institutions and dependent from personal motivation. This knowledge can help to improve MS rehabilitation programs.

## Data Sharing Statement

No additional data are available.

## Author Contributions

RL was involved in conception and design of study, acquisition of data, data analysis, interpretation of data, and drafting and revision of the article. SG was involved in conception and design of the study, in the data analysis, interpretation of data, and drafting of the article. E-MG was involved in interpretation of the data and drafting of the article. SS-M was involved in the acquisition of the data, data analysis, interpretation of the data, and drafting of the article. RW was involved in conception and design of the study, data analysis, interpretation of data, and drafting and revision of the article. The statistical analysis was done by SG and RL.

## Conflict of Interest Statement

The authors declare that the research was conducted in the absence of any commercial or financial relationships that could be construed as a potential conflict of interest.
