# Supplementary material for: Influence of Formal Education on Cognitive Reserve in Patients with Multiple Sclerosis
Source: Front Neurol. 2016 Mar 29;7:46. doi: 10.3389/fneur.2016.00046 (PMC4809897; doi:10.3389/fneur.2016.00046)
Supplement: Supplementary file 1 [file Table_1.PDF]

German tests	American version	German version
Wechsler Intellligenztest für Erwachsene WIE	Wechsler Adult Intelligence Scale	Aster von M, Neubauer A, Horn R: Wechsler Intelligenztest für Erwachsene WIE. 2. Auflage Pearson Assessment & Information GmbH, Frankfurt/M. 2009
California Verbal Learning Test CVLT	California Verbal Learning Test	Niemann H, Sturm W, Thöne-Otto AIT, Willmes K. California Verbal Learning Test. Deutschsprachige Adaptation. Pearson Assessment & Information GmbH, Frankfurt/M. 2008
Rey Visual Design Learning Test	Rey Visual Design Learning Test	no German version
Corsi blockspan	Corsi blockspan	no German version
Ruff 2&7	Ruff 2&7	no German version
Regensburger Wortflüssigkeitstest	Semantic fluency	Aschenbrenner S, Tucha O, Lange KW: Regensburger Wortflüssigkeitstest RWT Handanweisung. Hogrefe, Göttingen, Bern, Toronto, Seattle 2000
Controlled Oral Word Association Test	Controlled Oral Word Association Test	no German version
Trail Making Test A and B	Tombaugh TN. Trail Making Test A and B: normative data stratified by age and education. *Arch Clin Neuropsychol.* 2004 19:203-214	no German version
Rey Complex Figure Copy and Delay	Rey Complex Figure Copy and Delay	no German version
Wechsler Logical Memory I and II	Wechsler Logical Memory I and II	Wechsler Memory Scale-fourth edition (WMS-IV) Manual. NCS Pearson, Inc., Frankfurt/M. 2012
Alertness (Testbatterie zur Aufmerksamkeitsprüfung TAP)	only German version	Zimmermann P, Fimm B: Testbatterie zur Aufmerksamkeitsprüfung (TAP) Version 2.1 Psychologische Testsysteme, Herzogenrath 2007
